# Targeted protein degradation in CNS disorders: a promising route to novel therapeutics?

**DOI:** 10.3389/fnmol.2024.1370509

**Published:** 2024-04-15

**Authors:** Sandra Kuemper, Andrew G. Cairns, Kristian Birchall, Zhi Yao, Jonathan M. Large

**Affiliations:** LifeArc, Accelerator Building, Open Innovation Campus, Stevenage, United Kingdom

**Keywords:** CNS degraders, TPD, PROTAC, molecular glue, UPS, E3 ligase

## Abstract

Targeted protein degradation (TPD) is a rapidly expanding field, with various PROTACs (proteolysis-targeting chimeras) in clinical trials and molecular glues such as immunomodulatory imide drugs (IMiDs) already well established in the treatment of certain blood cancers. Many current approaches are focused on oncology targets, leaving numerous potential applications underexplored. Targeting proteins for degradation offers a novel therapeutic route for targets whose inhibition remains challenging, such as protein aggregates in neurodegenerative diseases. This mini review focuses on the prospect of utilizing TPD for neurodegenerative disease targets, particularly PROTAC and molecular glue formats and opportunities for novel CNS E3 ligases. Some key challenges of utilizing such modalities including molecular design of degrader molecules, drug delivery and blood brain barrier penetrance will be discussed.

## Introduction

**Figure 1:**
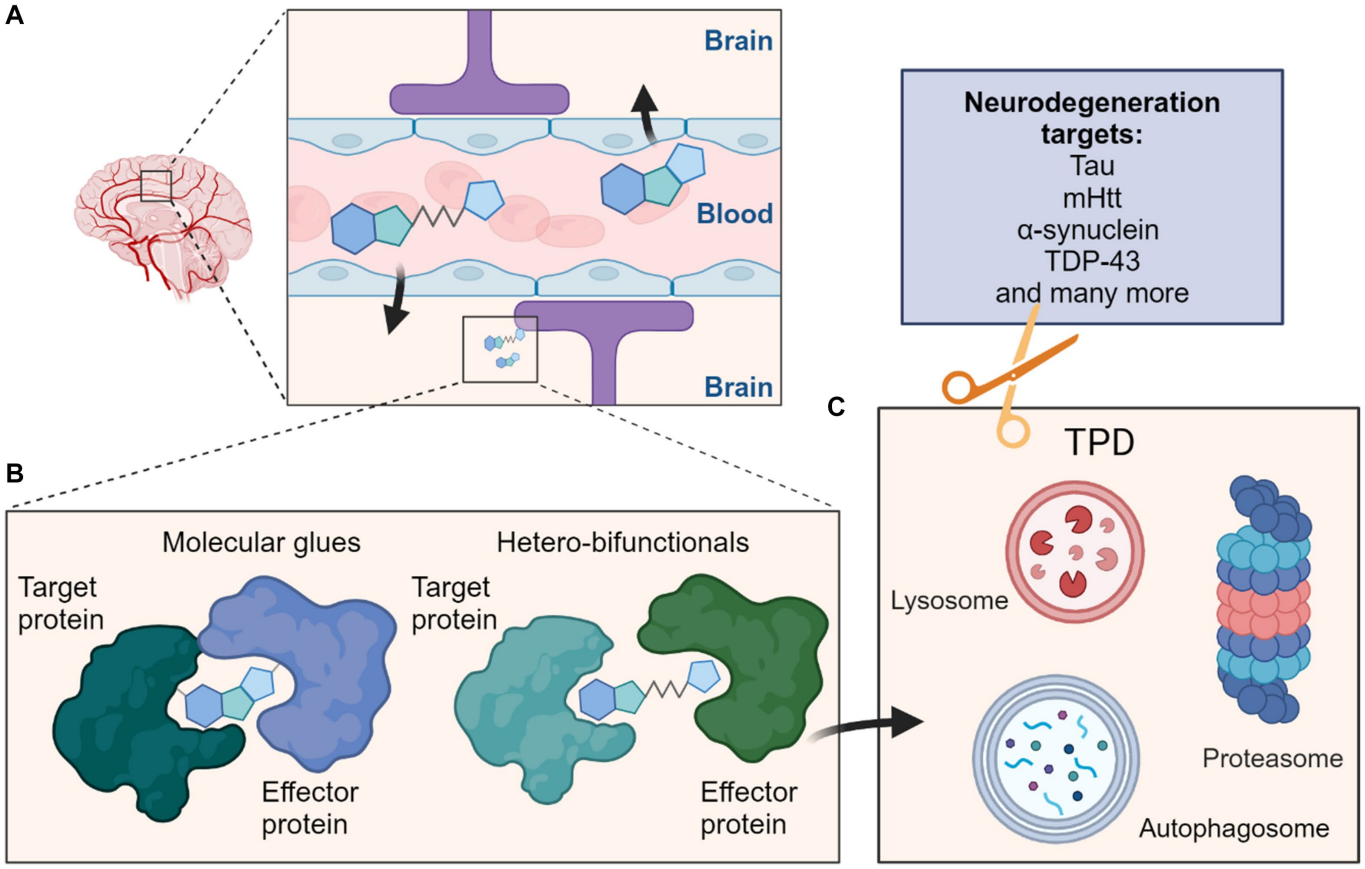
Schematic overview depicting targeted protein degradation approaches for neurodegeneration targets. **(A)** One major consideration for CNS-targeted ligands is the requirement for sufficient permeation of the blood–brain barrier (BBB); this is most often achieved via appropriate compound design and property metric strategies, although actively co-opting cross-BBB transport mechanisms remains an alternative possibility. **(B)** Such ligands could be MG typically of lower molecular weight which induce interactions between a target protein and effector protein, or the larger heterobifunctionals which classically contain ligands for both the target protein and effector protein connected by a linker. **(C)** These molecules can then induce protein degradation of the target protein by targeting them to cellular degradation machineries such as the ubiquitin proteasome system (UPS) or autophagy-lysosome pathways. Many neurodegeneration targets such as proteins prone to aggregation would be amenable to such strategies and their degradation may ameliorate effects of these diseases.

Targeted protein degradation (TPD) is a new modality with potential to drug poorly tractable targets. PROTAC (proteolysis-targeting chimera) or molecular glue (MG)-driven ternary complex formation with an E3 ligase utilizes cells’ ubiquitin-proteasome system (UPS) to degrade targets. Several such molecules have entered clinical development (Kong and Jones, 2023). Two E3 ligases, Von Hippel–Lindau (VHL) and Cereblon (CRBN), are regularly harnessed for therapeutic TPD approaches; both belong to the Cullin-RING E3 Ligase (CRL) family (Bondeson et al., 2015; Zengerle et al., 2015). Most current activity is in oncology, with indications including central nervous system (CNS)-associated pathologies less explored. Therapeutic development for CNS diseases is challenging due to blood brain barrier (BBB) permeability constraints, and in the druggability of protein aggregates which often characterize neurodegenerative pathologies. Commonly used degrader approaches have the potential to target proteins or aggregated complexes for degradation by the ubiquitin-proteasome (UPS) system (Bekes et al., 2022; Zhu et al., 2024) or autophagy-lysosome machinery (Pei et al., 2021; Ji et al., 2022). Targeted protein degraders act through event-driven pharmacology via non-reversible removal of functional components, and their potency is boosted by a catalytic mode of action (MOA), enabling sub-stoichiometric dosing regimens (Bekes et al., 2022). An extended pharmacokinetic (PK)- pharmacodynamic (PD) disconnect (Mares et al., 2020), can afford a reduction in off-target toxicity. These represent advantages over occupancy driven pharmacology often displayed by small molecule inhibitors. Further, degradation can be driven by ternary complex stability (Bondeson et al., 2018), for which protein–protein interaction (PPI) driven cooperativity is a key factor. As such, ligand-binding site affinity can be lower, potentially useful in targeting proteins without functional sites, such as the protein aggregates observed in neurodegenerative disease (Figure 1).

## PROTACs in CNS disease

Degradation approaches have been examined in proof-of-concept studies for neurodegenerative diseases (Thomas et al., 2023). Conceptually, protein or aggregates can be degraded if a selective small molecule binder is available. Utilizing degron tagging to induce proximity between a given target and an E3 ligase of interest may validate the approach (Ottis et al., 2017).

Many neurodegenerative disorders feature accumulation of aggregated proteins. Examples include Tauopathies, described in frontotemporal dementia (FTD) and Alzheimer’s disease (AD), and characterized by accumulation of aberrant Tau proteins (Morris et al., 2011; Gotz et al., 2013; Wang et al., 2014). Post-translational modifications of Tau lead to misfolding, mislocalization and oligomerization, resulting in neuronal toxicity (Gotz et al., 2013; Medina, 2018; Congdon et al., 2023). Studies utilizing a KEAP1-dependent peptide PROTAC showed degradation of intracellular Tau (Lu et al., 2018), providing evidence that Tau can be degraded by TPD approaches. Several studies report ternary complex formation and degradation of toxic forms of Tau using PROTACs to recruit the E3 ligases VHL or CRBN (Silva et al., 2019; Wang et al., 2021; Silva et al., 2022). A Tau-selective degrader QC-01-175, showed preferential degradation of aberrant Tau in FTD patient-derived neuronal cell models compared to healthy controls (Silva et al., 2019). A follow-up study optimized the linkers and demonstrated improved degradation of insoluble protein (Silva et al., 2022).

α-Synucleinopathies in Parkinson’s disease (PD) are also a potential degrader target. Abnormal accumulation in neurons leads to the formation of Lewy bodies and neurites, hallmarks of PD (Spillantini et al., 1997, 1998), and α-synuclein may disrupt normal lysosomal function (Bourdenx et al., 2014), suggesting degradation via the UPS as a possible approach. Utilizing an α-synuclein peptide and proteasome-targeting motif resulted in ubiquitination and degradation, attenuating neuronal toxicity (Qu et al., 2020). PROTACs based on three E3 ligase binders and α-synuclein aggregation inhibitor sery384, which binds oligomeric α-synuclein (Wagner et al., 2013; Pena-Diaz et al., 2023), induced degradation of aggregated α-synuclein in an overexpression system using preformed fibrils (Wen et al., 2023). The efficacy of these molecules was low and PROTAC MOA experiments are required to determine the value of this approach.

Mutant huntingtin (mHTT) aggregation is thought to cause neuronal injury and apoptosis in Huntington’s disease (Fiorillo et al., 2021). One approach to targeting mHTT for degradation linked amyloid binding imaging agents (Olsen et al., 2010; Matsumura et al., 2012) to IAP binders and showed degradation of HTT and mHTT in cells (Tomoshige et al., 2017, 2018).

Amyotrophic Lateral Sclerosis (ALS) and FTD show formation of cytoplasmic TAR DNA-binding protein (TDP-43) aggregates (Jo et al., 2020). In healthy cells, TDP-43 is predominantly nuclear and involved in transcriptional and post-transcriptional regulation and pre-mRNA splicing (Jo et al., 2020). A proportion of TDP-43 shuttles between the cytoplasm and nucleus as part of cellular stress responses. In ALS pathology, TDP-43 is found in insoluble cytoplasmic aggregates where it is often hyperphosphorylated, ubiquitinated and fragmented (Neumann et al., 2006; Hasegawa et al., 2008). Loss of nuclear TDP-43 into cytoplasmic aggregates could be a driver of ALS pathology (Ling et al., 2013). PROTACs linking aggregate binders, benzothiazole-aniline derivatives (BTA), to Pomalidomide, degraded overexpressed truncated c-terminal TDP-43, attenuating the reduction in cell viability (Tseng et al., 2023). Both varying expression and subcellular localization of target proteins would offer opportunities for selectivity, giving spatiotemporal control of therapeutic activity.

## PROTAC molecular design constraints

Constraining small molecule property design space within thresholds improves toxicity and PKPD outcomes in later development (Lipinski et al., 2001; Wager et al., 2010; Shultz, 2019). CNS-targeted PROTACs are large molecules which also require BBB penetration, and as such occupy several restrictive and complex design spaces. Property considerations are stricter for CNS-targeted ligands requiring passive BBB permeation (Wager et al., 2016). A detailed analysis of CNS metrics is beyond the scope of this review, but consideration of the widely used Pfizer CNS MPO metric (Wager et al., 2010, 2016) favors chemotypes of moderate size and limited polarity (total polar surface area, TPSA, LogD, hydrogen bond donors, HBD), a pattern reproduced in the B3DB compound database with annotated BBB permeation characteristics (Meng et al., 2021).

PROTACs are also large molecules which extend into challenging non-traditional, “beyond rule of 5” (bRo5) property space (Matsson et al., 2016; Volak et al., 2023). There are additional resulting challenges: (i) larger molecules can behave differently especially regarding permeability and transport (Guimaraes et al., 2012; Doak et al., 2014; Matsson et al., 2016; Matsson and Kihlberg, 2017; Pye et al., 2017; Linker et al., 2023); (ii) available chemical space is larger; (iii) large molecules are often more conformationally complex (Rossi Sebastiano et al., 2018; Mobitz, 2023); (iv) fewer molecules from bRo5 space have been studied; (v) those studied molecules cluster in niches (e.g., macrocycles) and represent a less even distribution (Doak et al., 2014). Metric-based approaches nonetheless exist (Degoey et al., 2018; Poongavanam et al., 2018; Mobitz, 2023). Broadly, these suggest that higher LogP values are tolerated; greater ligand complexity could compensate for lipophilicity driven promiscuity (Hann et al., 2001; Doak et al., 2016; Matsson et al., 2016; Egbert et al., 2019). This may compensate for size associated permeability challenges (Pye et al., 2017; Pike et al., 2020). With increasing size, TPSA becomes less representative of ligand polarity, and conformational changes which modulate exposed polarity in response to environment (chameleonic behavior) become accessible (Rossi Sebastiano et al., 2018; Ono et al., 2021; Garcia Jimenez et al., 2023). While the extent to which this behavior is observed in bRo5 chemical matter is debated (Mobitz, 2023), accessible conformations of significantly lower polarity than suggested by TPSA values may be required (Whitty et al., 2016; Mobitz, 2023). Tight restrictions on hydrogen bonding are suggested (Doak et al., 2014; Matsson et al., 2016; Whitty et al., 2016; Mobitz, 2023; Volak et al., 2023).

Analysis of PROTAC chemotypes shows these molecules broadly comply with bRo5 paradigms (Edmondson et al., 2019), although stricter limits on HBD were observed in rat absorption data (Hornberger and Araujo, 2023). A CNS penetrant PROTAC must balance sufficient polarity to provide specific target engagement and solubility while crossing the BBB (Doak et al., 2014). Additionally, increased efflux is common in bRo5 (Doak et al., 2014; Matsson et al., 2016; Cantrill et al., 2020; Cox et al., 2023) and PROTAC development space (Klein et al., 2021). Beyond CNS considerations, the combination of differentiated property space (in particular lipophilicity) and pharmacology results in additional considerations around absorption, distribution, metabolism, and excretion (ADME) testing (Cantrill et al., 2020; Pike et al., 2020; Apprato et al., 2023; Hornberger and Araujo, 2023; Volak et al., 2023).

**Figure 2:**
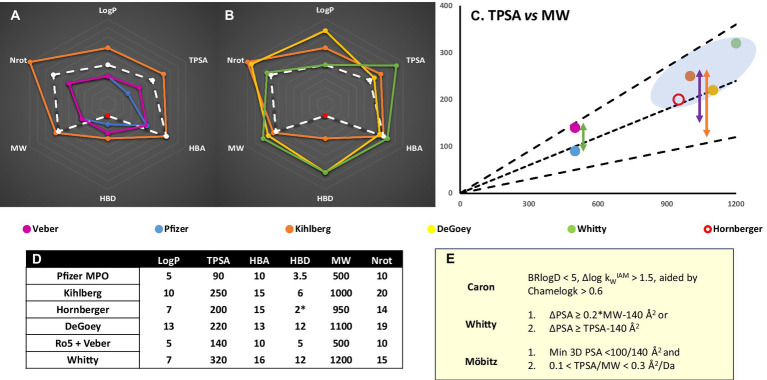
**(A)** Chemical space representation for CNS (Pfizer MPO) *vs* Ro5 (Lipinski et al., 2001; Veber et al., 2002) bRo5 (Doak et al., 2014; Villar et al., 2014; Poongavanam et al., 2018) and PROTAC guidelines (Hornberger and Araujo, 2023) for oral absorption. Red data point refers to unsatisfied HBD – intramolecular hydrogen bond pairs not counted. MGD designs would be expected to fall within Ro5/CNS metric space. **(B)** PROTAC specific values *vs* different metrics or limitations identified from bRo5 space studies (Degoey et al., 2018). **(C)** Several TPSA guidelines **(E)** scaled by MW; (Whitty et al., 2016; Mobitz, 2023) black lines are TPSA = 0.1, 0.2, 0.3*MW, plotted points are the MW and TPSA outer limits of metric sets from **(A,B)**. The green arrow shows the TPSA difference between Ro5 and CNS Ro5 space. The purple arrow represents the TPSA *vs* minimum/3D PSA difference seemingly required for bRo5 oral absorption. BBB penetration will impose stricter minimum/3D PSA limitations and a correspondingly larger ΔPSA is likely necessary (orange). **(D)** Numerical values plotted in **(A,B)** for different metric spaces. **(E).** Polarity based chameleonicity or ΔPSA/3D TPSA metrics defined in the literature (Whitty et al., 2016; Garcia Jimenez et al., 2023; Mobitz, 2023).

Lastly, heterobifunctional molecules display behaviors not attributable to a single component, including different target specificity to their POI ligands, whose affinity does not predict ternary complex stability or productivity (Bondeson et al., 2018). Cooperative binding behavior can arise from induced PPI (Song et al., 2021). Given the limitations implied by the combination of CNS permeation, bRo5 space and efflux liabilities, the ‘budget’ of polar contacts and hydrogen bonds available for the POI binder alone is limited (Hughes et al., 2021; Hornberger and Araujo, 2023). The interactions induced in ternary complex formation can generate contact areas equivalent to those in other bRo5 modalities (Doak and Kihlberg, 2017; Bondeson et al., 2018). Considered alongside the importance of conformational masking of polarity in bRo5 space, property-based linker design and rigidification (Troup et al., 2020; Klein et al., 2021; Poongavanam et al., 2022), and holistic consideration of molecular properties will prove vital. The combination of CNS and PROTAC constraints will require greater chameleonicity and cooperativity (Figure 2), and each component to adopt multiple roles to achieve sufficient permeability and binding specificity.

Several CNS penetrant PROTACs have been reported, utilizing VHL (Wang et al., 2021; Liu et al., 2022) and CRBN binders (Mihalic, 2023); oral exposure was demonstrated for each (Liu et al., 2022; Mihalic, 2023). All display low Kp,uu values (Loryan et al., 2022), but nonetheless several demonstrate *in vivo* activity (Wang et al., 2021; Mihalic, 2023). These observations may reflect exposure requirements depending on potency and event *vs* occupancy driven pharmacology (Volak et al., 2023). Notably the clinical candidate NX-5948 minimizes size and hydrogen bonding consistent with earlier considerations (Mihalic, 2023). Though development of PROTACs for neurodegenerative diseases will have to meet the challenges of BBB and brain penetrance, there is considerable commercial activity in employing this therapeutic strategy (Kargbo, 2020).

## Expanding the repertoire of E3 ligases

Two key E3 ligases have been routinely harnessed for TPD approaches. CRBN ligands dominate PROTACs and MG in clinical stages (Kong and Jones, 2023), mainly due to their size and “drug-like” properties, although the risk of teratogenicity remains a concern (Smith and Mitchell, 2018). Two VHL-based PROTACs, namely BCL-X_L_ degrader DT2216 (Khan et al., 2019) and KRAS^G12D^ degrader ASP-3082 (Nagashima et al., 2022) are in clinical trials, and E7820, which glues RBM39 to the DCAF15 ligase complex (Chirnomas et al., 2023; Kong and Jones, 2023). Other E3 ligases such as RNF114 (Spradlin et al., 2019; Luo et al., 2021), KEAP1 (Wei et al., 2021; Du et al., 2022), DCAF11 (Zhang et al., 2021; Xue et al., 2023) and DCAF16 (Zhang et al., 2019) have been harnessed for TPD, but not progressed beyond pre-clinical investigations and their effectiveness in degrading a wide range of targets remains unclear. Several of these E3s are ubiquitously expressed, likely leading to target degradation in multiple tissues increasing toxicity when targeting essential proteins. The use of tissue enriched or selective E3 ligases as PROTAC or molecular glue degrader (MGD) targets has huge potential for diseases restricted to one tissue or organ such as CNS pathologies. One clinical example is DT2116, which degrades BCL-X_L_. BCL-X_L_ inhibition causes on-target and dose-limiting thrombocytopenia (Kaefer et al., 2014); however, the PROTAC showed significantly lower toxicity due to low VHL expression in platelets (Khan et al., 2019). CNS enriched E3s may allow CNS target degradation while limiting potential toxicity in unwanted tissues. Examples include RNF182 identified as a brain-enriched E3 upregulated in AD patients (Liu et al., 2008). Several E3s of the Kelch family such as ENC1, KBTBD11, KLHL2, KLHL32, KLHL35 and KLHL4 also have preferential brain expression (Ehrlich et al., 2020), where other family members such as KEAP1 have already been utilized in PROTAC-based degraders (Wei et al., 2021). Two TRIM-family E3 Ligases (TRIM9 and TRIM67) are neuronally enriched and regulate neuronal morphological changes (Tanji et al., 2010; Menon et al., 2015; Boyer et al., 2020). A related E3 Ligase TRIM21 shows proximity-induced degradation using the “Trim-away” technique, degrading targets through binding the Fc region of antibodies (Clift et al., 2017). Developments in specific fragment libraries (Whitehurst et al., 2023) and covalent approaches have been investigated for E3 ligand finding (Tao et al., 2022; Belcher et al., 2023; Toriki et al., 2023), but not demonstrated in CNS space. Advances in understanding which E3 ligases could be utilized for TPD purposes (Liu et al., 2023) will support efforts to discover and develop new E3 ligase ligands (Ishida and Ciulli, 2021).

## Molecular glue degraders (MGDs) for CNS disease

Most MGDs facilitate degradation via induced protein–protein interactions between an E3 ligase and a target (Rui et al., 2023). Historically, the discovery of MG and their mechanism was retrospective. For example, the severe birth defects caused by thalidomide (Vargesson, 2015) are thought to be caused by degradation of the neosubstrate transcription factor SALL4 via thalidomide binding to the E3 ligase CRBN (Ito et al., 2010) The landscape of neosubstrates depends on ligand structure (Matyskiela et al., 2016; Nowak et al., 2023; Szewczyk et al., 2024), unlocking opportunities for novel target discovery. The aryl sulfonamide Indisulam blocks cell cycle progression (Owa et al., 1999) through degradation of mRNA splicing factor RBM39 mediated by recruitment of the E3 ligase DCAF15 (Han et al., 2017). Indisulam and derivatives do not display high affinity to either target protein; ternary complex formation results in nanomolar affinities (Du et al., 2019). Linking aryl sulfonamides to the BET bromodomain inhibitor JQ1 demonstrated that BRD4 degradation was independent of DCAF15 and driven by stabilizing existing interactions of adjacent bromodomains with the E3 ligase DCAF16 (Hsia et al., 2024). These intramolecular bivalent glues highlight another mechanistic possibility, demonstrating the pharmacological scope of these approaches and the importance of evaluating off-target degradation profiles of novel degraders.

Efforts are underway to rationalize the discovery of MG. One approach is the E3 ligase centric generation of compound libraries, currently focused on CRBN-binding chemotypes (Powell et al., 2020) but which will extend to other E3s upon ligand and neosubstrate discovery. Another is to engineer cell lines to overexpress, eliminate, conformationally trap or inactivate a given E3 ligase; then screen libraries for their ability to degrade targets (Mayor-Ruiz et al., 2019; Hanzl et al., 2023; Ng et al., 2023). However, CNS relevant cell types are often more difficult to manipulate than cancer cell lines. DNA-encoded library (DEL) screening has been adapted to screen for ternary complex formation (Mason et al., 2023) and in combination with disease relevant recombinant aggregates or proteins would be a definite starting point for CNS relevant MG identification. Opportunities for novel MG identification depend on an understanding of interactions between relevant proteins. Techniques such as Bio-ID/Turbo-ID can identify potential E3 ligase substrates and weak protein–protein interactions (Branon et al., 2018; Barroso-Gomila et al., 2023) in disease relevant cell types as a precursor to DEL screening for novel MG discovery. Matching an E3 ligase to a target protein through CRISPR-based degron mapping would further annotate pre-selection (Timms et al., 2023; Zhang et al., 2023) to identify CNS-enriched E3s and target pairings.

Structure-based design of MG remains an enticing but challenging prospect. Modeling or obtaining structural information on ternary complexes is difficult due to their complexity (Casement et al., 2021). Artificial Intelligence (AI) and Machine Learning (ML) approaches have driven advances in protein structure prediction, but their utility in predicting protein–protein complexes remains uncertain (Burke et al., 2023), although improvements in accuracy within classes may be realized as more structural data is acquired.

## Glue molecular design considerations

A CNS-targeted MG would generally comprise a small molecule requiring BBB permeability; although differences in binding (Cao et al., 2022; Sasso et al., 2023) and the specifics of event driven pharmacology (Riching et al., 2022) may create distinctions. MG chemotype diversity (e.g., thalidomide *vs* rapamycin), and scaffold over-representation complicate definition of generic design constraints (Dong et al., 2021; Sasso et al., 2023) although it appears that most non-natural product MG can achieve their function within Ro5 parameter space (Dong et al., 2021; Dewey et al., 2023). Re-positioning existing chemotypes is common in glue development- (Guo et al., 2019; Geiger et al., 2022; Sasso et al., 2023; Toriki et al., 2023), so scaffold specific considerations may be widely applicable.

The most studied MGD class are immunomodulatory imide drugs (IMiD) analogues, which bind to CRBN (Cao et al., 2022) via structures related to biological signaling motifs (Ichikawa et al., 2022) and engage a recurring POI motif represented in numerous targets, largely via hydrophobic groups (Oleinikovas et al., 2024). Recent work on parameter optimization for CRBN ligand specificity (Szewczyk et al., 2024) suggested significant target dependence, but reduction in aromatic carbocycles, introduction of heteroatoms and increased spatial complexity appear beneficial. LogP or TPSA were not determinative, while increasing HBD count was detrimental in several classes. This suggests that CRBN MGD *selectivity* optimization is not necessarily in tension with CNS property space. However, the determinative power of these MPOs was weak across much of the chemical space surveyed. The observation that CRBN ligands extend the PPI surface (Oleinikovas et al., 2024), and can cause PPI relevant conformational changes (Watson et al., 2022) is consistent with groups apparently distal from the common binding site influencing substrate selectivity (Nowak et al., 2023; Nguyen et al., 2024). In studies of CRBN binders it was observed that more proteins interact than are degraded (Sievers et al., 2018) an effect which may give rise to stronger selectivity in cells (Oleinikovas et al., 2024).

Among other known degrader classes, Cyclin K degraders have been shown to function throughout the Ro5 LogP and MW range, with a hydrophobic contact and ligand modification of the PPI again noted as key considerations in binding and selectivity (Kozicka et al., 2023). Indisulam analogues have less binary affinity and bind in a less conserved site but are involved in extensive interface interactions which modify the resulting PPI surfaces (Faust et al., 2020). From the limited studies to date the influence of conformational and PPI effects such as extending or remodeling surfaces and steric exclusion appear to be considerations for the efficacy and selectivity of glue interactions, which may influence the choices made in glue design (Geiger et al., 2022; Kozicka et al., 2023; Jiang et al., 2024; Oleinikovas et al., 2024).

In practical terms non-natural product MG fall mostly within chemical space for which existing ADME assays are effective. Compound efficiency metrics, however, may need to be re-defined to adequately discriminate between early stage ligands (Jia et al., 2024).

## Non E3-ligase driven degradation

Strategies to target proteins or aggregates to the autophagy-lysosome machinery are less well understood than UPS-targeting degraders. For example, microarray-based screening led to the discovery of mHTT–light chain 3 (LC3) binders which induced autophagy-mediated degradation of mHTT (Li et al., 2019). The underlying MOA has since been questioned as it was found that the indolinone LC3 binders covalently bind to the E3 Ligase DCAF11, leading to UPS-driven degradation (Xue et al., 2023). α-Synuclein has been targeted to the lysosomal proteolytic machinery by peptides containing α-synuclein-binding, membrane-penetrating and chaperone-mediated autophagy-targeting motifs (Tong et al., 2023). α-Synuclein has also been degraded by macroautophagy, employing an autophagy-targeting chimera (AUTOTAC) binding both α-Synuclein aggregates and autophagy receptor p62/SQSTM1/Sequestosome-1 (Lee et al., 2023). Strategies to clear extracellular proteins or aggregates include lysosome-targeting chimeras (LYTACs), which are bifunctional molecules binding both a cell-surface lysosome-shuttling receptor and the target of interest (Banik et al., 2020), which could be especially valuable for neurodegenerative diseases characterized by toxic insoluble extracellular aggregates. Some of these techniques may lead to the activation of autophagy having an added benefit, since mutations in genes encoding regulators of the autophagy and UPS machineries, such as p62/SQSTM1, VCP or Ubiquilin-2 are often found in FTD or ALS and are thought to cause downregulation of autophagy/proteasomal functions (Deng et al., 2017). Further, protein aggregates or oligomers may interfere with proteasomal function, resulting in lower PROTAC efficacy. One study describes allosteric impairment of the 20S proteasome substrate gate by soluble oligomers composed of either amyloid-β (Aβ) 1–42, α-synuclein, or mHTT, preventing substrates from entering the 19S pore (Thibaudeau et al., 2018). It is not known whether these oligomers inhibit the proteasome sufficiently to prevent efficient degradation.

## Concluding remarks and outlook

Several proof-of-concept studies have been published on the degradation of known neurodegenerative disease targets. Key challenges remain in the discovery of selective small molecules suitable for degradation approaches and their development towards therapeutic applications in CNS space. For heterobifunctional molecules such as PROTACs, the most important aspects center on complex mechanistic and property design considerations. By contrast, MG discovery space particularly requires identification of degraders and the PPI which they enable. For both contexts, novel CNS-enriched E3 ligase enablement will drive progress in this area. Despite these challenges, the event driven pharmacology allowed by degraders may be especially suited to CNS disease targets, allowing for innovative therapeutic developments in an area of significant unmet medical need.

## Author contributions

SK: Conceptualization, Visualization, Writing – original draft, Writing – review & editing. AC: Conceptualization, Writing – original draft, Writing – review & editing. KB: Writing – original draft, Writing – review & editing. ZY: Writing – original draft, Writing – review & editing. JL: Writing – original draft, Writing – review & editing.

## References

[ref1] Apprato G. Ermondi G. Caron G. (2023). The quest for Oral Protac drugs: evaluating the weaknesses of the screening pipeline. ACS Med. Chem. Lett. 14, 879–883. doi: 10.1021/acsmedchemlett.3c00231, PMID: 37465314 PMC10351046

[ref2] Banik S. M. Pedram K. Wisnovsky S. Ahn G. Riley N. M. Bertozzi C. R. (2020). Lysosome-targeting chimaeras for degradation of extracellular proteins. Nature 584, 291–297. doi: 10.1038/s41586-020-2545-9, PMID: 32728216 PMC7727926

[ref3] Barroso-Gomila O. Merino-Cacho L. Muratore V. Perez C. Taibi V. Maspero E.. (2023). BioE3 identifies specific substrates of ubiquitin E3 ligases. Nat. Commun. 14:7656. doi: 10.1038/s41467-023-43326-8, PMID: 37996419 PMC10667490

[ref4] Bekes M. Langley D. R. Crews C. M. (2022). Protac targeted protein degraders: the past is prologue. Nat. Rev. Drug Discov. 21, 181–200. doi: 10.1038/s41573-021-00371-6, PMID: 35042991 PMC8765495

[ref5] Belcher B. P. Ward C. C. Nomura D. K. (2023). Ligandability of E3 ligases for targeted protein degradation applications. Biochemistry 62, 588–600. doi: 10.1021/acs.biochem.1c00464, PMID: 34473924 PMC8928483

[ref6] Bondeson D. P. Mares A. Smith I. E. Ko E. Campos S. Miah A. H.. (2015). Catalytic in vivo protein knockdown by small-molecule Protacs. Nat. Chem. Biol. 11, 611–617. doi: 10.1038/nchembio.1858, PMID: 26075522 PMC4629852

[ref7] Bondeson D. P. Smith B. E. Burslem G. M. Buhimschi A. D. Hines J. Jaime-Figueroa S.. (2018). Lessons in Protac design from selective degradation with a promiscuous warhead. Cell. Chem. Biol. 25, 78–87.e5. doi: 10.1016/j.chembiol.2017.09.010 29129718 PMC5777153

[ref8] Bourdenx M. Bezard E. Dehay B. (2014). Lysosomes and alpha-synuclein form a dangerous duet leading to neuronal cell death. Front. Neuroanat. 8:83. doi: 10.3389/fnana.2014.00083 25177278 PMC4132369

[ref9] Boyer N. P. Mccormick L. E. Menon S. Urbina F. L. Gupton S. L. (2020). A pair of E3 ubiquitin ligases compete to regulate filopodial dynamics and axon guidance. J. Cell Biol. 219:e201902088. doi: 10.1083/jcb.201902088, PMID: 31820781 PMC7039193

[ref10] Branon T. C. Bosch J. A. Sanchez A. D. Udeshi N. D. Svinkina T. Carr S. A.. (2018). Efficient proximity labeling in living cells and organisms with Turboid. Nat. Biotechnol. 36, 880–887. doi: 10.1038/nbt.4201, PMID: 30125270 PMC6126969

[ref11] Burke D. F. Bryant P. Barrio-Hernandez I. Memon D. Pozzati G. Shenoy A.. (2023). Towards a structurally resolved human protein interaction network. Nat. Struct. Mol. Biol. 30, 216–225. doi: 10.1038/s41594-022-00910-8, PMID: 36690744 PMC9935395

[ref12] Cantrill C. Chaturvedi P. Rynn C. Petrig Schaffland J. Walter I. Wittwer M. B. (2020). Fundamental aspects of Dmpk optimization of targeted protein degraders. Drug Discov. Today 25, 969–982. doi: 10.1016/j.drudis.2020.03.012, PMID: 32298797

[ref13] Cao S. Kang S. Mao H. Yao J. Gu L. Zheng N. (2022). Defining molecular glues with a dual-nanobody cannabidiol sensor. Nat. Commun. 13:815. doi: 10.1038/s41467-022-28507-1, PMID: 35145136 PMC8831599

[ref14] Casement R. Bond A. Craigon C. Ciulli A. (2021). Mechanistic and structural features of Protac ternary complexes. Methods Mol. Biol. 2365, 79–113. doi: 10.1007/978-1-0716-1665-9_5, PMID: 34432240

[ref15] Chirnomas D. Hornberger K. R. Crews C. M. (2023). Protein degraders enter the clinic - a new approach to cancer therapy. Nat. Rev. Clin. Oncol. 20, 265–278. doi: 10.1038/s41571-023-00736-3 36781982 PMC11698446

[ref16] Clift D. Mcewan W. A. Labzin L. I. Konieczny V. Mogessie B. James L. C.. (2017). A method for the acute and rapid degradation of endogenous proteins. Cell 171, 1692–1706.e18. doi: 10.1016/j.cell.2017.10.033 29153837 PMC5733393

[ref17] Congdon E. E. Ji C. Tetlow A. M. Jiang Y. Sigurdsson E. M. (2023). Tau-targeting therapies for Alzheimer disease: current status and future directions. Nat. Rev. Neurol. 19, 715–736. doi: 10.1038/s41582-023-00883-2, PMID: 37875627 PMC10965012

[ref18] Cox B. Nicolai J. Williamson B. (2023). The role of the efflux transporter, P-glycoprotein, at the blood-brain barrier in drug discovery. Biopharm. Drug Dispos. 44, 113–126. doi: 10.1002/bdd.2331, PMID: 36198662

[ref19] Degoey D. A. Chen H. J. Cox P. B. Wendt M. D. (2018). Beyond the rule of 5: lessons Learned from AbbVie's drugs and compound collection. J. Med. Chem. 61, 2636–2651. doi: 10.1021/acs.jmedchem.7b00717, PMID: 28926247

[ref20] Deng Z. Sheehan P. Chen S. Yue Z. (2017). Is amyotrophic lateral sclerosis/frontotemporal dementia an autophagy disease? Mol. Neurodegener. 12:90. doi: 10.1186/s13024-017-0232-6, PMID: 29282133 PMC5746010

[ref21] Dewey J. A. Delalande C. Azizi S. A. Lu V. Antonopoulos D. Babnigg G. (2023). Molecular glue discovery: current and future approaches. J. Med. Chem. 66, 9278–9296. doi: 10.1021/acs.jmedchem.3c00449, PMID: 37437222 PMC10805529

[ref22] Doak B. C. Kihlberg J. (2017). Drug discovery beyond the rule of 5 - opportunities and challenges. Expert Opin. Drug Discov. 12, 115–119. doi: 10.1080/17460441.2017.1264385 27883294

[ref23] Doak B. C. Over B. Giordanetto F. Kihlberg J. (2014). Oral druggable space beyond the rule of 5: insights from drugs and clinical candidates. Chem. Biol. 21, 1115–1142. doi: 10.1016/j.chembiol.2014.08.013, PMID: 25237858

[ref24] Doak B. C. Zheng J. Dobritzsch D. Kihlberg J. (2016). How beyond rule of 5 drugs and clinical candidates bind to their targets. J. Med. Chem. 59, 2312–2327. doi: 10.1021/acs.jmedchem.5b01286, PMID: 26457449

[ref25] Dong G. Ding Y. He S. Sheng C. (2021). Molecular glues for targeted protein degradation: from serendipity to rational discovery. J. Med. Chem. 64, 10606–10620. doi: 10.1021/acs.jmedchem.1c00895, PMID: 34319094

[ref26] Du G. Jiang J. Henning N. J. Safaee N. Koide E. Nowak R. P.. (2022). Exploring the target scope of Keap1 E3 ligase-based Protacs. Cell. Chem. Biol. 29, 1470–1481.e31. doi: 10.1016/j.chembiol.2022.08.003 36070758 PMC9588736

[ref27] Du X. Volkov O. A. Czerwinski R. M. Tan H. Huerta C. Morton E. R.. (2019). Structural basis and kinetic pathway of Rbm39 recruitment to Dcaf15 by a sulfonamide molecular glue E7820. Structure 27, 1625–1633.e3. doi: 10.1016/j.str.2019.10.005 31693911

[ref28] Edmondson S. D. Yang B. Fallan C. (2019). Proteolysis targeting chimeras (Protacs) in 'beyond rule-of-five' chemical space: recent progress and future challenges. Bioorg. Med. Chem. Lett. 29, 1555–1564. doi: 10.1016/j.bmcl.2019.04.030, PMID: 31047748

[ref29] Egbert M. Whitty A. Keseru G. M. Vajda S. (2019). Why some targets benefit from beyond rule of five drugs. J. Med. Chem. 62, 10005–10025. doi: 10.1021/acs.jmedchem.8b01732, PMID: 31188592 PMC7102492

[ref30] Ehrlich K. C. Baribault C. Ehrlich M. (2020). Epigenetics of muscle- and brain-specific expression of Klhl family genes. Int. J. Mol. Sci. 21:8394. doi: 10.3390/ijms21218394, PMID: 33182325 PMC7672584

[ref31] Faust T. B. Yoon H. Nowak R. P. Donovan K. A. Li Z. Cai Q.. (2020). Structural complementarity facilitates E7820-mediated degradation of Rbm39 by Dcaf15. Nat. Chem. Biol. 16, 7–14. doi: 10.1038/s41589-019-0378-3, PMID: 31686031 PMC6917914

[ref32] Fiorillo A. Morea V. Colotti G. Ilari A. (2021). Huntingtin ubiquitination mechanisms and novel possible therapies to decrease the toxic effects of mutated huntingtin. J. Pers. Med. 11:1309. doi: 10.3390/jpm11121309, PMID: 34945781 PMC8709430

[ref33] Garcia Jimenez D. Vallaro M. Rossi Sebastiano M. Apprato G. D'agostini G. Rossetti P.. (2023). Chamelogk: A chromatographic Chameleonicity quantifier to design orally bioavailable beyond-rule-of-5 drugs. J. Med. Chem. 66, 10681–10693. doi: 10.1021/acs.jmedchem.3c00823, PMID: 37490408 PMC10424176

[ref34] Geiger T. M. Schäfer S. C. Dreizler J. K. Walz M. Hausch F. (2022). Clues to molecular glues. Curr. Chem. Biol. 2:100018. doi: 10.1016/j.crchbi.2021.100018

[ref35] Gotz J. Xia D. Leinenga G. Chew Y. L. Nicholas H. (2013). What Renders Tau Toxic. Front. Neurol. 4:72. doi: 10.3389/fneur.2013.00072 23772223 PMC3677143

[ref36] Guimaraes C. R. Mathiowetz A. M. Shalaeva M. Goetz G. Liras S. (2012). Use of 3D properties to characterize beyond rule-of-5 property space for passive permeation. J. Chem. Inf. Model. 52, 882–890. doi: 10.1021/ci300010y, PMID: 22394163

[ref37] Guo Z. Hong S. Y. Wang J. Rehan S. Liu W. Peng H.. (2019). Rapamycin-inspired macrocycles with new target specificity. Nat. Chem. 11, 254–263. doi: 10.1038/s41557-018-0187-4 30532015 PMC6435255

[ref38] Han T. Goralski M. Gaskill N. Capota E. Kim J. Ting T. C.. (2017). Anticancer sulfonamides target splicing by inducing Rbm39 degradation via recruitment to Dcaf15. Science 356:eaal3755. doi: 10.1126/science.aal3755, PMID: 28302793

[ref39] Hann M. M. Leach A. R. Harper G. (2001). Molecular complexity and its impact on the probability of finding leads for drug discovery. J. Chem. Inf. Comput. Sci. 41, 856–864. doi: 10.1021/ci000403i, PMID: 11410068

[ref40] Hanzl A. Barone E. Bauer S. Yue H. Nowak R. P. Hahn E.. (2023). E3-specific degrader discovery by dynamic tracing of substrate receptor abundance. J. Am. Chem. Soc. 145, 1176–1184. doi: 10.1021/jacs.2c10784, PMID: 36602777 PMC9853857

[ref41] Hasegawa M. Arai T. Nonaka T. Kametani F. Yoshida M. Hashizume Y.. (2008). Phosphorylated Tdp-43 in frontotemporal lobar degeneration and amyotrophic lateral sclerosis. Ann. Neurol. 64, 60–70. doi: 10.1002/ana.21425, PMID: 18546284 PMC2674108

[ref42] Hornberger K. R. Araujo E. M. V. (2023). Physicochemical property determinants of Oral absorption for Protac protein degraders. J. Med. Chem. 66, 8281–8287. doi: 10.1021/acs.jmedchem.3c00740, PMID: 37279490 PMC10291545

[ref43] Hsia O. Hinterndorfer M. Cowan A. D. Iso K. Ishida T. Sundaramoorthy R.. (2024). Targeted protein degradation via intramolecular bivalent glues. Nature 627, 204–211. doi: 10.1038/s41586-024-07089-6, PMID: 38383787 PMC10917667

[ref44] Hughes S. J. Testa A. Thompson N. Churcher I. (2021). The rise and rise of protein degradation: opportunities and challenges ahead. Drug Discov. Today 26, 2889–2897. doi: 10.1016/j.drudis.2021.08.006, PMID: 34419629

[ref45] Ichikawa S. Flaxman H. A. Xu W. Vallavoju N. Lloyd H. C. Wang B.. (2022). The E3 ligase adapter cereblon targets the C-terminal cyclic imide degron. Nature 610, 775–782. doi: 10.1038/s41586-022-05333-5, PMID: 36261529 PMC10316063

[ref46] Ishida T. Ciulli A. (2021). E3 ligase ligands for Protacs: how they were found and how to discover new ones. Slas. Discov. 26, 484–502. doi: 10.1177/2472555220965528, PMID: 33143537 PMC8013866

[ref47] Ito T. Ando H. Suzuki T. Ogura T. Hotta K. Imamura Y.. (2010). Identification of a primary target of thalidomide teratogenicity. Science 327, 1345–1350. doi: 10.1126/science.1177319, PMID: 20223979

[ref48] Ji C. H. Kim H. Y. Lee M. J. Heo A. J. Park D. Y. Lim S.. (2022). The Autotac chemical biology platform for targeted protein degradation via the autophagy-lysosome system. Nat. Commun. 13:904. doi: 10.1038/s41467-022-28520-4, PMID: 35173167 PMC8850458

[ref49] Jia L. Weiss D. Shields B. Claus B. Shanmugasundaram V. Johnson S.. (2024). Scoring methods in lead optimization of molecular glues. ChemRxiv. doi: 10.26434/chemrxiv-2023-4hn4s-v2

[ref50] Jiang W. Jiang Y. Luo Y. Qiao W. Yang T. (2024). Facilitating the development of molecular glues: opportunities from serendipity and rational design. Eur. J. Med. Chem. 263:115950. doi: 10.1016/j.ejmech.2023.115950 37984298

[ref51] Jo M. Lee S. Jeon Y. M. Kim S. Kwon Y. Kim H. J. (2020). The role of Tdp-43 propagation in neurodegenerative diseases: integrating insights from clinical and experimental studies. Exp. Mol. Med. 52, 1652–1662. doi: 10.1038/s12276-020-00513-7, PMID: 33051572 PMC8080625

[ref52] Kaefer A. Yang J. Noertersheuser P. Mensing S. Humerickhouse R. Awni W.. (2014). Mechanism-based pharmacokinetic/pharmacodynamic meta-analysis of navitoclax (Abt-263) induced thrombocytopenia. Cancer Chemother. Pharmacol. 74, 593–602. doi: 10.1007/s00280-014-2530-9 25053389

[ref53] Kargbo R. B. (2020). Protac compounds targeting alpha-Synuclein protein for treating Neurogenerative disorders: Alzheimer's and Parkinson's diseases. ACS Med. Chem. Lett. 11, 1086–1087. doi: 10.1021/acsmedchemlett.0c00192, PMID: 32550983 PMC7294559

[ref54] Khan S. Zhang X. Lv D. Zhang Q. He Y. Zhang P.. (2019). A selective Bcl-X(L) Protac degrader achieves safe and potent antitumor activity. Nat. Med. 25, 1938–1947. doi: 10.1038/s41591-019-0668-z, PMID: 31792461 PMC6898785

[ref55] Klein V. G. Bond A. G. Craigon C. Lokey R. S. Ciulli A. (2021). Amide-to-Ester substitution as a strategy for optimizing Protac permeability and cellular activity. J. Med. Chem. 64, 18082–18101. doi: 10.1021/acs.jmedchem.1c01496, PMID: 34881891 PMC8713283

[ref56] Kong N. R. Jones L. H. (2023). Clinical translation of targeted protein degraders. Clin. Pharmacol. Ther. 114, 558–568. doi: 10.1002/cpt.2985 37399310

[ref57] Kozicka Z. Suchyta D. J. Focht V. Kempf G. Petzold G. Jentzsch M.. (2023). Design principles for cyclin K molecular glue degraders. Nat. Chem. Biol. 20, 93–102. doi: 10.1038/s41589-023-01409-z 37679459 PMC10746543

[ref58] Lee J. Sung K. W. Bae E. J. Yoon D. Kim D. Lee J. S.. (2023). Targeted degradation of ⍺-synuclein aggregates in Parkinson's disease using the Autotac technology. Mol. Neurodegener. 18:41. doi: 10.1186/s13024-023-00630-7, PMID: 37355598 PMC10290391

[ref59] Li Z. Wang C. Wang Z. Zhu C. Li J. Sha T.. (2019). Allele-selective lowering of mutant Htt protein by Htt-Lc3 linker compounds. Nature 575, 203–209. doi: 10.1038/s41586-019-1722-1, PMID: 31666698

[ref60] Ling S. C. Polymenidou M. Cleveland D. W. (2013). Converging mechanisms in Als and Ftd: disrupted Rna and protein homeostasis. Neuron 79, 416–438. doi: 10.1016/j.neuron.2013.07.033, PMID: 23931993 PMC4411085

[ref61] Linker S. M. Schellhaas C. Kamenik A. S. Veldhuizen M. M. Waibl F. Roth H. J.. (2023). Lessons for Oral bioavailability: how Conformationally flexible cyclic peptides enter and cross lipid membranes. J. Med. Chem. 66, 2773–2788. doi: 10.1021/acs.jmedchem.2c01837, PMID: 36762908 PMC9969412

[ref62] Lipinski C. A. Lombardo F. Dominy B. W. Feeney P. J. (2001). Experimental and computational approaches to estimate solubility and permeability in drug discovery and development settings. Adv. Drug Deliv. Rev. 46, 3–26. doi: 10.1016/S0169-409X(00)00129-0, PMID: 11259830

[ref63] Liu X. Kalogeropulou A. F. Domingos S. Makukhin N. Nirujogi R. S. Singh F.. (2022). Discovery of Xl01126: A potent, fast, cooperative, selective, orally bioavailable, and blood-brain barrier penetrant Protac degrader of leucine-rich repeat kinase 2. J. Am. Chem. Soc. 144, 16930–16952. doi: 10.1021/jacs.2c05499, PMID: 36007011 PMC9501899

[ref64] Liu Q. Y. Lei J. X. Sikorska M. Liu R. (2008). A novel brain-enriched E3 ubiquitin ligase Rnf182 is up regulated in the brains of Alzheimer's patients and targets Atp6V0C for degradation. Mol. Neurodegener. 3:4. doi: 10.1186/1750-1326-3-4, PMID: 18298843 PMC2279130

[ref65] Liu Y. Yang J. Wang T. Luo M. Chen Y. Chen C.. (2023). Expanding Protactable genome universe of E3 ligases. Nat. Commun. 14:6509. doi: 10.1038/s41467-023-42233-2, PMID: 37845222 PMC10579327

[ref66] Loryan I. Reichel A. Feng B. Bundgaard C. Shaffer C. Kalvass C.. (2022). Unbound brain-to-plasma partition coefficient, Kp,uu,brain—a game changing parameter for CNS drug discovery and development. Pharm. Res. 39, 1321–1341. doi: 10.1007/s11095-022-03246-6, PMID: 35411506 PMC9246790

[ref67] Lu M. Liu T. Jiao Q. Ji J. Tao M. Liu Y.. (2018). Discovery of a Keap1-dependent peptide Protac to knockdown tau by ubiquitination-proteasome degradation pathway. Eur. J. Med. Chem. 146, 251–259. doi: 10.1016/j.ejmech.2018.01.063, PMID: 29407955

[ref68] Luo M. Spradlin J. N. Boike L. Tong B. Brittain S. M. Mckenna J. M.. (2021). Chemoproteomics-enabled discovery of covalent Rnf114-based degraders that mimic natural product function. Cell. Chem. Biol. 28, 559–566.e15. doi: 10.1016/j.chembiol.2021.01.005 33513350 PMC8052289

[ref01] Mares A. Miah A. H. Smith I. E. D. Rackham M. Thawani A. R. Cryan J.. (2020). Extended pharmacodynamic responses observed upon PROTAC-mediated degradation of RIPK2. Commun. Biol. 3:140. doi: 10.1038/s42003-020-0868-6 32198438 PMC7083851

[ref69] Mason J. W. Chow Y. T. Hudson L. Tutter A. Michaud G. Westphal M. V.. (2023). DNA-encoded library-enabled discovery of proximity-inducing small molecules. Nat. Chem. Biol. 20, 170–179. doi: 10.1038/s41589-023-01458-4 37919549 PMC10917151

[ref70] Matsson P. Doak B. C. Over B. Kihlberg J. (2016). Cell permeability beyond the rule of 5. Adv. Drug Deliv. Rev. 101, 42–61. doi: 10.1016/j.addr.2016.03.013, PMID: 27067608

[ref71] Matsson P. Kihlberg J. (2017). How big is too big for cell permeability? J. Med. Chem. 60, 1662–1664. doi: 10.1021/acs.jmedchem.7b00237, PMID: 28234469

[ref72] Matsumura K. Ono M. Kimura H. Ueda M. Nakamoto Y. Togashi K.. (2012). (18)F-labeled phenyldiazenyl benzothiazole for in vivo imaging of neurofibrillary tangles in Alzheimer's disease brains. ACS Med. Chem. Lett. 3, 58–62. doi: 10.1021/ml200230e, PMID: 24900371 PMC4025873

[ref73] Matyskiela M. E. Lu G. Ito T. Pagarigan B. Lu C. C. Miller K.. (2016). A novel cereblon modulator recruits Gspt1 to the Crl4(Crbn) ubiquitin ligase. Nature 535, 252–257. doi: 10.1038/nature18611 27338790

[ref74] Mayor-Ruiz C. Jaeger M. G. Bauer S. Brand M. Sin C. Hanzl A.. (2019). Plasticity of the Cullin-Ring ligase repertoire shapes sensitivity to ligand-induced protein degradation. Mol. Cell 75, 849–858.e8. 31442425 10.1016/j.molcel.2019.07.013

[ref75] Medina M. (2018). An overview on the clinical development of tau-based therapeutics. Int. J. Mol. Sci. 19:1160. doi: 10.3390/ijms19041160, PMID: 29641484 PMC5979300

[ref76] Meng F. Xi Y. Huang J. Ayers P. W. (2021). A curated diverse molecular database of blood-brain barrier permeability with chemical descriptors. Sci. Data 8:289. doi: 10.1038/s41597-021-01069-5, PMID: 34716354 PMC8556334

[ref77] Menon S. Boyer N. P. Winkle C. C. Mcclain L. M. Hanlin C. C. Pandey D.. (2015). The E3 ubiquitin ligase Trim9 is a Filopodia off switch required for netrin-dependent axon guidance. Dev. Cell 35, 698–712. doi: 10.1016/j.devcel.2015.11.022, PMID: 26702829 PMC4707677

[ref78] Mihalic J. (2023) First disclosure of Nx-5948, an Oral targeted degrader of Bruton's tyrosine kinase (Btk) for the treatment of B-cell malignancies. American Chemical Society conference - first time disclosures.

[ref79] Mobitz H. (2023). Design principles for balancing lipophilicity and permeability in beyond rule of 5 space. ChemMedChem 19:e202300395. doi: 10.1002/cmdc.202300395 37986275

[ref80] Morris M. Maeda S. Vossel K. Mucke L. (2011). The many faces of tau. Neuron 70, 410–426. doi: 10.1016/j.neuron.2011.04.009, PMID: 21555069 PMC3319390

[ref81] Nagashima T. Inamura K. Nishizono Y. Suzuki A. Tanaka H. Yoshinari T.. (2022). 85 (Pb075) - Asp3082, a first-in-class novel Kras G12D degrader, exhibits remarkable anti-tumor activity in Kras G12D mutated cancer models. Eur. J. Cancer 174:S30. doi: 10.1016/S0959-8049(22)00881-4

[ref82] Neumann M. Sampathu D. M. Kwong L. K. Truax A. C. Micsenyi M. C. Chou T. T.. (2006). Ubiquitinated Tdp-43 in frontotemporal lobar degeneration and amyotrophic lateral sclerosis. Science 314, 130–133. doi: 10.1126/science.1134108 17023659

[ref83] Ng A. Offensperger F. Cisneros J. A. Scholes N. S. Malik M. Villanti L.. (2023). Discovery of molecular glue degraders via isogenic morphological profiling. ACS Chem. Biol. 18, 2464–2473. doi: 10.1021/acschembio.3c00598, PMID: 38098458 PMC10764104

[ref84] Nguyen T. M. Sreekanth V. Deb A. Kokkonda P. Tiwari P. K. Donovan K. A.. (2024). Proteolysis-targeting chimeras with reduced off-targets. Nat. Chem. 16, 218–228. doi: 10.1038/s41557-023-01379-8, PMID: 38110475 PMC10913580

[ref85] Nowak R. P. Che J. Ferrao S. Kong N. R. Liu H. Zerfas B. L.. (2023). Structural rationalization of Gspt1 and Ikzf1 degradation by thalidomide molecular glue derivatives. Rsc Med. Chem. 14, 501–506. doi: 10.1039/D2MD00347C, PMID: 36970148 PMC10034078

[ref86] Oleinikovas V. Gainza P. Ryckmans T. Fasching B. Thoma N. H. (2024). From thalidomide to rational molecular glue Design for Targeted Protein Degradation. Annu. Rev. Pharmacol. Toxicol. 64, 291–312. doi: 10.1146/annurev-pharmtox-022123-104147, PMID: 37585660

[ref87] Olsen J. S. Brown C. Capule C. C. Rubinshtein M. Doran T. M. Srivastava R. K.. (2010). Amyloid-binding small molecules efficiently block Sevi (semen-derived enhancer of virus infection)- and semen-mediated enhancement of Hiv-1 infection. J. Biol. Chem. 285, 35488–35496. doi: 10.1074/jbc.M110.163659, PMID: 20833717 PMC2975173

[ref88] Ono S. Naylor M. R. Townsend C. E. Okumura C. Okada O. Lee H. W.. (2021). Cyclosporin A: conformational complexity and Chameleonicity. J. Chem. Inf. Model. 61, 5601–5613. doi: 10.1021/acs.jcim.1c00771, PMID: 34672629 PMC9531541

[ref89] Ottis P. Toure M. Cromm P. M. Ko E. Gustafson J. L. Crews C. M. (2017). Assessing different E3 ligases for small molecule induced protein ubiquitination and degradation. ACS Chem. Biol. 12, 2570–2578. doi: 10.1021/acschembio.7b00485, PMID: 28767222

[ref90] Owa T. Yoshino H. Okauchi T. Yoshimatsu K. Ozawa Y. Sugi N. H.. (1999). Discovery of novel antitumor sulfonamides targeting G1 phase of the cell cycle. J. Med. Chem. 42, 3789–3799. doi: 10.1021/jm9902638, PMID: 10508428

[ref91] Pei J. Pan X. Wang A. Shuai W. Bu F. Tang P.. (2021). Developing potent Lc3-targeting Autac tools for protein degradation with selective autophagy. Chem. Commun. (Camb.) 57, 13194–13197. doi: 10.1039/D1CC04661F, PMID: 34816823

[ref92] Pena-Diaz S. Garcia-Pardo J. Ventura S. (2023). Development of small molecules targeting alpha-Synuclein aggregation: A promising strategy to treat Parkinson's disease. Pharmaceutics 15:839. doi: 10.3390/pharmaceutics15030839, PMID: 36986700 PMC10059018

[ref93] Pike A. Williamson B. Harlfinger S. Martin S. Mcginnity D. F. (2020). Optimising proteolysis-targeting chimeras (Protacs) for oral drug delivery: a drug metabolism and pharmacokinetics perspective. Drug Discov. Today 25, 1793–1800. doi: 10.1016/j.drudis.2020.07.013, PMID: 32693163

[ref94] Poongavanam V. Atilaw Y. Siegel S. Giese A. Lehmann L. Meibom D.. (2022). Linker-dependent folding rationalizes Protac cell permeability. J. Med. Chem. 65, 13029–13040. doi: 10.1021/acs.jmedchem.2c00877, PMID: 36170570 PMC9574858

[ref95] Poongavanam V. Doak B. C. Kihlberg J. (2018). Opportunities and guidelines for discovery of orally absorbed drugs in beyond rule of 5 space. Curr. Opin. Chem. Biol. 44, 23–29. doi: 10.1016/j.cbpa.2018.05.010 29803972

[ref96] Powell C. E. Du G. Che J. He Z. Donovan K. A. Yue H.. (2020). Selective degradation of Gspt1 by Cereblon modulators identified via a focused combinatorial library. ACS Chem. Biol. 15, 2722–2730. doi: 10.1021/acschembio.0c00520, PMID: 32865967 PMC7843009

[ref97] Pye C. R. Hewitt W. M. Schwochert J. Haddad T. D. Townsend C. E. Etienne L.. (2017). Nonclassical size dependence of permeation defines bounds for passive adsorption of large drug molecules. J. Med. Chem. 60, 1665–1672. doi: 10.1021/acs.jmedchem.6b01483, PMID: 28059508 PMC5677520

[ref98] Qu J. Ren X. Xue F. He Y. Zhang R. Zheng Y.. (2020). Specific knockdown of alpha-Synuclein by peptide-directed proteasome degradation rescued its associated neurotoxicity. Cell. Chem. Biol. 27, 751–762.e4. doi: 10.1016/j.chembiol.2020.03.010 32359427

[ref99] Riching K. M. Caine E. A. Urh M. Daniels D. L. (2022). The importance of cellular degradation kinetics for understanding mechanisms in targeted protein degradation. Chem. Soc. Rev. 51, 6210–6221. doi: 10.1039/D2CS00339B, PMID: 35792307

[ref100] Rossi Sebastiano M. Doak B. C. Backlund M. Poongavanam V. Over B. Ermondi G.. (2018). Impact of dynamically exposed polarity on permeability and solubility of chameleonic drugs beyond the rule of 5. J. Med. Chem. 61, 4189–4202. doi: 10.1021/acs.jmedchem.8b00347, PMID: 29608068

[ref101] Rui H. Ashton K. S. Min J. Wang C. Potts P. R. (2023). Protein-protein interfaces in molecular glue-induced ternary complexes: classification, characterization, and prediction. Rsc Chem. Biol. 4, 192–215. doi: 10.1039/D2CB00207H, PMID: 36908699 PMC9994104

[ref102] Sasso J. M. Tenchov R. Wang D. Johnson L. S. Wang X. Zhou Q. A. (2023). Molecular glues: the adhesive connecting targeted protein degradation to the clinic. Biochemistry 62, 601–623. doi: 10.1021/acs.biochem.2c00245, PMID: 35856839 PMC9910052

[ref103] Shultz M. D. (2019). Two decades under the influence of the rule of five and the changing properties of approved Oral drugs. J. Med. Chem. 62, 1701–1714. doi: 10.1021/acs.jmedchem.8b00686 30212196

[ref104] Sievers Q. L. Petzold G. Bunker R. D. Renneville A. Slabicki M. Liddicoat B. J.. (2018). Defining the human C2H2 zinc finger degrome targeted by thalidomide analogs through Crbn. Science 362:eaat0572. doi: 10.1126/science.aat0572, PMID: 30385546 PMC6326779

[ref105] Silva M. C. Ferguson F. M. Cai Q. Donovan K. A. Nandi G. Patnaik D.. (2019). Targeted degradation of aberrant tau in frontotemporal dementia patient-derived neuronal cell models. eLife 8:e45457. doi: 10.7554/eLife.45457, PMID: 30907729 PMC6450673

[ref106] Silva M. C. Nandi G. Donovan K. A. Cai Q. Berry B. C. Nowak R. P.. (2022). Discovery and optimization of tau targeted protein degraders enabled by patient induced pluripotent stem cells-derived neuronal models of Tauopathy. Front. Cell. Neurosci. 16:801179. doi: 10.3389/fncel.2022.801179 35317195 PMC8934437

[ref107] Smith R. L. Mitchell S. C. (2018). Thalidomide-type teratogenicity: structure-activity relationships for congeners. Toxicol. Res. (Camb) 7, 1036–1047. doi: 10.1039/c8tx00187a, PMID: 30542600 PMC6248049

[ref108] Song J. H. Wagner N. D. Yan J. Li J. Huang R. Y. Balog A. J.. (2021). Native mass spectrometry and gas-phase fragmentation provide rapid and in-depth topological characterization of a Protac ternary complex. Cell. Chem. Biol. 28, 1528–1538.e4. doi: 10.1016/j.chembiol.2021.05.005 34081921 PMC8592818

[ref109] Spillantini M. G. Crowther R. A. Jakes R. Hasegawa M. Goedert M. (1998). Alpha-Synuclein in filamentous inclusions of Lewy bodies from Parkinson's disease and dementia with lewy bodies. Proc. Natl. Acad. Sci. USA 95, 6469–6473. doi: 10.1073/pnas.95.11.6469 9600990 PMC27806

[ref110] Spillantini M. G. Schmidt M. L. Lee V. M. Trojanowski J. Q. Jakes R. Goedert M. (1997). Alpha-synuclein in Lewy bodies. Nature 388, 839–840. doi: 10.1038/42166 9278044

[ref111] Spradlin J. N. Hu X. Ward C. C. Brittain S. M. Jones M. D. Ou L.. (2019). Harnessing the anti-cancer natural product nimbolide for targeted protein degradation. Nat. Chem. Biol. 15, 747–755. doi: 10.1038/s41589-019-0304-8, PMID: 31209351 PMC6592714

[ref112] Szewczyk S. M. Verma I. Edwards J. T. Weiss D. R. Chekler E. L. P. (2024). Trends in Neosubstrate degradation by Cereblon-based molecular glues and the development of novel multiparameter optimization scores. J. Med. Chem. 67, 1327–1335. doi: 10.1021/acs.jmedchem.3c01872 38170610

[ref113] Tanji K. Kamitani T. Mori F. Kakita A. Takahashi H. Wakabayashi K. (2010). Trim9, a novel brain-specific E3 ubiquitin ligase, is repressed in the brain of Parkinson's disease and dementia with Lewy bodies. Neurobiol. Dis. 38, 210–218. doi: 10.1016/j.nbd.2010.01.007, PMID: 20085810 PMC2942959

[ref114] Tao Y. Remillard D. Vinogradova E. V. Yokoyama M. Banchenko S. Schwefel D.. (2022). Targeted protein degradation by electrophilic Protacs that Stereoselectively and site-specifically engage Dcaf1. J. Am. Chem. Soc. 144, 18688–18699. doi: 10.1021/jacs.2c08964, PMID: 36170674 PMC10347610

[ref115] Thibaudeau T. A. Anderson R. T. Smith D. M. (2018). A common mechanism of proteasome impairment by neurodegenerative disease-associated oligomers. Nat. Commun. 9:1097. doi: 10.1038/s41467-018-03509-0, PMID: 29545515 PMC5854577

[ref116] Thomas B. A. I. Lewis H. L. Jones D. H. Ward S. E. (2023). Central nervous system targeted protein degraders. Biomol. Ther. 13:1164. doi: 10.3390/biom13081164, PMID: 37627229 PMC10452695

[ref117] Timms R. T. Mena E. L. Leng Y. Li M. Z. Tchasovnikarova I. A. Koren I.. (2023). Defining E3 ligase-substrate relationships through multiplex Crispr screening. Nat. Cell Biol. 25, 1535–1545. doi: 10.1038/s41556-023-01229-2, PMID: 37735597 PMC10567573

[ref118] Tomoshige S. Nomura S. Ohgane K. Hashimoto Y. Ishikawa M. (2017). Discovery of small molecules that induce the degradation of huntingtin. Angew. Chem. Int. Ed. Engl. 56, 11530–11533. doi: 10.1002/anie.201706529, PMID: 28703441

[ref119] Tomoshige S. Nomura S. Ohgane K. Hashimoto Y. Ishikawa M. (2018). Degradation of huntingtin mediated by a hybrid molecule composed of Iap antagonist linked to phenyldiazenyl benzothiazole derivative. Bioorg. Med. Chem. Lett. 28, 707–710. doi: 10.1016/j.bmcl.2018.01.012, PMID: 29366651

[ref120] Tong Y. Zhu W. Chen J. Zhang W. Xu F. Pang J. (2023). Targeted degradation of alpha-Synuclein by autophagosome-anchoring chimera peptides. J. Med. Chem. 66, 12614–12628. doi: 10.1021/acs.jmedchem.3c01303, PMID: 37652467

[ref121] Toriki E. S. Papatzimas J. W. Nishikawa K. Dovala D. Frank A. O. Hesse M. J.. (2023). Rational chemical Design of Molecular Glue Degraders. Acs Cent. Sci. 9, 915–926. doi: 10.1021/acscentsci.2c01317, PMID: 37252349 PMC10214506

[ref122] Troup R. I. Fallan C. Baud M. G. J. (2020). Current strategies for the design of Protac linkers: a critical review. Explor Target Antitumor. Ther. 1, 273–312. doi: 10.37349/etat.2020.00018, PMID: 36046485 PMC9400730

[ref123] Tseng Y. L. Lu P. C. Lee C. C. He R. Y. Huang Y. A. Tseng Y. C.. (2023). Degradation of neurodegenerative disease-associated Tdp-43 aggregates and oligomers via a proteolysis-targeting chimera. J. Biomed. Sci. 30:27. doi: 10.1186/s12929-023-00921-7, PMID: 37101169 PMC10131537

[ref124] Vargesson N. (2015). Thalidomide-induced teratogenesis: history and mechanisms. Birth Defects Res. C Embryo Today 105, 140–156. doi: 10.1002/bdrc.21096, PMID: 26043938 PMC4737249

[ref125] Veber D. F. Johnson S. R. Cheng H. Y. Smith B. R. Ward K. W. Kopple K. D. (2002). Molecular properties that influence the oral bioavailability of drug candidates. J. Med. Chem. 45, 2615–2623. doi: 10.1021/jm020017n, PMID: 12036371

[ref126] Villar E. A. Beglov D. Chennamadhavuni S. Porco J. A. Jr. Kozakov D. Vajda S.. (2014). How proteins bind macrocycles. Nat. Chem. Biol. 10, 723–731. doi: 10.1038/nchembio.1584, PMID: 25038790 PMC4417626

[ref127] Volak L. P. Duevel H. M. Humphreys S. Nettleton D. Phipps C. Pike A.. (2023). Industry perspective on the pharmacokinetic and absorption, distribution, metabolism, and excretion characterization of heterobifunctional protein degraders. Drug Metab. Dispos. 51, 792–803. doi: 10.1124/dmd.122.001154, PMID: 37041086

[ref128] Wager T. T. Hou X. Verhoest P. R. Villalobos A. (2010). Moving beyond rules: the development of a central nervous system multiparameter optimization (Cns Mpo) approach to enable alignment of druglike properties. ACS Chem. Neurosci. 1, 435–449. doi: 10.1021/cn100008c, PMID: 22778837 PMC3368654

[ref129] Wager T. T. Hou X. Verhoest P. R. Villalobos A. (2016). Central nervous system multiparameter optimization desirability: application in drug discovery. ACS Chem. Neurosci. 7, 767–775. doi: 10.1021/acschemneuro.6b00029, PMID: 26991242

[ref130] Wagner J. Ryazanov S. Leonov A. Levin J. Shi S. Schmidt F.. (2013). Anle138b: a novel oligomer modulator for disease-modifying therapy of neurodegenerative diseases such as prion and Parkinson's disease. Acta Neuropathol. 125, 795–813. doi: 10.1007/s00401-013-1114-9, PMID: 23604588 PMC3661926

[ref131] Wang J. Z. Gao X. Wang Z. H. (2014). The physiology and pathology of microtubule-associated protein tau. Essays Biochem. 56, 111–123. doi: 10.1042/bse0560111, PMID: 25131590

[ref132] Wang W. Zhou Q. Jiang T. Li S. Ye J. Zheng J.. (2021). A novel small-molecule Protac selectively promotes tau clearance to improve cognitive functions in Alzheimer-like models. Theranostics 11, 5279–5295. doi: 10.7150/thno.55680 33859747 PMC8039949

[ref133] Watson E. R. Novick S. Matyskiela M. E. Chamberlain P. P. A. H. D. L. P. Zhu J. Tran E.. (2022). Molecular glue CelmoD compounds are regulators of cereblon conformation. Science 378, 549–553. doi: 10.1126/science.add7574, PMID: 36378961 PMC9714526

[ref134] Wei J. Meng F. Park K. S. Yim H. Velez J. Kumar P.. (2021). Harnessing the E3 ligase Keap1 for targeted protein degradation. J. Am. Chem. Soc. 143, 15073–15083. doi: 10.1021/jacs.1c04841, PMID: 34520194 PMC8480205

[ref135] Wen T. Chen J. Zhang W. Pang J. (2023). Design, synthesis and biological evaluation of alpha-Synuclein proteolysis-targeting chimeras. Molecules 28:4458. doi: 10.3390/molecules28114458, PMID: 37298935 PMC10254247

[ref136] Whitehurst B. C. Bauer M. R. Edfeldt F. Gunnarsson A. Margreitter C. Rawlins P. B.. (2023). Design and evaluation of a low hydrogen Bond donor count fragment screening set to aid hit generation of Protacs intended for Oral delivery. J. Med. Chem. 66, 7594–7604. doi: 10.1021/acs.jmedchem.3c00493 37224440

[ref137] Whitty A. Zhong M. Viarengo L. Beglov D. Hall D. R. Vajda S. (2016). Quantifying the chameleonic properties of macrocycles and other high-molecular-weight drugs. Drug Discov. Today 21, 712–717. doi: 10.1016/j.drudis.2016.02.005, PMID: 26891978 PMC5821503

[ref138] Xue G. Xie J. Hinterndorfer M. Cigler M. Dotsch L. Imrichova H.. (2023). Discovery of a drug-like, natural product-inspired Dcaf11 ligand Chemotype. Nat. Commun. 14:7908. doi: 10.1038/s41467-023-43657-6 38036533 PMC10689823

[ref139] Zengerle M. Chan K. H. Ciulli A. (2015). Selective small molecule induced degradation of the bet Bromodomain protein Brd4. ACS Chem. Biol. 10, 1770–1777. doi: 10.1021/acschembio.5b00216, PMID: 26035625 PMC4548256

[ref140] Zhang X. Crowley V. M. Wucherpfennig T. G. Dix M. M. Cravatt B. F. (2019). Electrophilic Protacs that degrade nuclear proteins by engaging Dcaf16. Nat. Chem. Biol. 15, 737–746. doi: 10.1038/s41589-019-0279-5, PMID: 31209349 PMC6592777

[ref141] Zhang X. Luukkonen L. M. Eissler C. L. Crowley V. M. Yamashita Y. Schafroth M. A.. (2021). Dcaf11 supports targeted protein degradation by electrophilic proteolysis-targeting chimeras. J. Am. Chem. Soc. 143, 5141–5149. doi: 10.1021/jacs.1c00990, PMID: 33783207 PMC8309050

[ref142] Zhang Z. Sie B. Chang A. Leng Y. Nardone C. Timms R. T.. (2023). Elucidation of E3 ubiquitin ligase specificity through proteome-wide internal degron mapping. Mol. Cell 83, 3377–3392.e6. doi: 10.1016/j.molcel.2023.08.022 37738965 PMC10594193

[ref143] Zhu W. Zhang W. Chen J. Tong Y. Xu F. Pang J. (2024). Discovery of effective dual Protac degraders for neurodegenerative disease-associated aggregates. J. Med. Chem. 67, 3448–3466. doi: 10.1021/acs.jmedchem.3c01719 38356330

